# Progesterone Concentrations during Canine Pregnancy

**DOI:** 10.3390/ani11123369

**Published:** 2021-11-24

**Authors:** Janna Hinderer, Julia Lüdeke, Lisa Riege, Peggy Haimerl, Alexander Bartel, Barbara Kohn, Corinna Weber, Elisabeth Müller, Sebastian P. Arlt

**Affiliations:** 1 Faculty of Veterinary Medicine, Clinic for Animal Reproduction, Freie Universität Berlin, 14163 Berlin, Germany; janna.hinderer@fu-berlin.de (J.H.); julia.luedeke@fu-berlin.de (J.L.); lisa.riege@fu-berlin.de (L.R.); haimerl@btkberlin.de (P.H.); 2 Faculty of Veterinary Medicine, Institute for Veterinary Epidemiology and Biostatistics, Freie Universität Berlin, 14163 Berlin, Germany; alexander.bartel@fu-berlin.de; 3 Faculty of Veterinary Medicine, Clinic for Small Animals, Freie Universität Berlin, 14163 Berlin, Germany; barbara.kohn@fu-berlin.de; 4 Laboklin GmbH & Co KG, 97688 Bad Kissingen, Germany; weber@laboklin.com (C.W.); mueller@laboklin.com (E.M.)

**Keywords:** dog, pregnancy, progesterone, hypoluteoidism, parturition, litter size, gestation length

## Abstract

**Simple Summary:**

For breeders and bitches alike, pregnancy is a challenging period. Insufficient serum progesterone concentrations have frequently been suggested to be responsible for pregnancy loss or early parturition, without any scientific evidence to support those claims. In this study, a large number of bitches was followed throughout pregnancy, and serum progesterone concentrations were determined to deduce which concentrations could be considered normal. Three blood samples were collected, one each during early, mid and late pregnancy. The results indicated that progesterone concentrations can be lower than previously described as adequate in the veterinary literature, with the bitch still exhibiting no signs of pregnancy distress and giving birth to healthy puppies at the expected time.

**Abstract:**

Pregnancy and lactation are amongst the most challenging times of a bitch’s life. Most studies focusing on the endocrinological aspect of pregnancy consider only a small number of animals. The aim of this study was to evaluate progesterone (P4) concentrations in a large number of bitches during early, mid and late pregnancy. In total, 126 bitches of various breeds were recruited following a thorough clinical and gynecological examination during estrus. Blood samples were collected three times (T1–T3) during pregnancy or from non-pregnant dogs in diestrus, and P4 was measured via chemiluminescence. At T1 (11–19 days post-ovulation (dpo)), serum P4 concentrations were 30.23 ± 6.65 ng/mL and 28.45 ± 6.26 ng/mL, at T2 (23–32 dpo) they were 22.73 ± 6.27 ng/mL and 22.59 ± 5.77 ng/mL and at T3 (52–60 dpo) they were 6.68 ± 2.18 ng/mL and 3.17 ± 2.26 ng/mL, in pregnant (*n* = 98) and non-pregnant (*n* = 23) dogs respectively. The P4 concentrations differed significantly between pregnant and non-pregnant animals at the last examination (*p* ≤ 0.001). In the context of hypoluteoidism, the gathered data yielded interesting results. Overall, 28 out of 98 pregnant bitches showed a greater decline (>15 ng/mL) in P4 concentrations from early to mid-pregnancy, and 56 bitches showed P4 concentrations lower than deemed adequate (>20 ng/mL at T1 and T2, >5 ng/mL at T3) according to existing recommendations. Despite not being supplemented with P4, none of those animals suffered from abortion or preterm delivery. Considering that supplementation of P4 can entail considerable risks for the bitch and the puppies, more research on P4 concentration patterns, diagnosis of hypoluteoidism and treatment indications and options is indicated.

## 1. Introduction

Progesterone (P4) is a steroid hormone belonging to the group of progestogens. With its manifold effects on the body, most notably on the uterus, it is essential for the establishment and maintenance of pregnancy. Contrary to some other animal species, in which the placenta is involved in hormone synthesis towards the end of pregnancy, P4 only originates from the corpora lutea in the bitch [1,2,3]. The serum concentration of P4 during pregnancy has been subject of various articles published in the past 50 years [4,5,6]. However, most of the studies performed so far only examined a limited number of bitches and focused on specific breeds (see Table 1).

A related field of research is hypoluteoidism (or hypoluteinism) in the bitch, i.e., a primary failure of sufficient P4 secretion by the corpora lutea during pregnancy, which usually causes embryonic resorption or abortion of the whole litter. A premature decline in P4 secretion, most notably a decline below 2 ng/mL for more than 24 h, results in increased uterine contractility and cervical dilation, leading to the inability of the uterus to maintain pregnancy [7,8]. However, these conclusions about hypoluteoidism are based on research involving the administration of luteolytic drugs and subsequent hormone measurements. The reason for a premature decline in P4 in seemingly healthy bitches remains unclear, even though the amount of research concerning this subject has increased in the past 15 years [6,9,10,11]. Previous findings suggest insufficient production of luteotropic agents, such as relaxin and/or prolactin, which would consequently lead to insufficient P4 secretion in late pregnancy [12]. Other studies suggest malfunctions within the ovaries as the primary cause [13]. Furthermore, an occurrence of P4 antibodies in up to 20% of pregnant bitches has been found and discussed as a possible factor in the pathogenesis of hypoluteoidism [14]. The reported cumulation of suspected cases or an increased risk for hypoluteoidism in certain breeds (i.e., primarily German Shepherd) indicates a genetic component [15,16]. There is, however, no further research published that examines this possibility. Most authors agree that the diagnosis of hypoluteoidism can be achieved by eliminating all other possible causes for pregnancy loss, especially infectious causes such as Brucella canis, other opportunistic vaginal bacteria that may cause uterine infection, Canine herpesvirus or systemic diseases [17,18]. Other non-infectious causes include trauma, inadequate nutrition or genetic abnormalities in the offspring [19].

It has been claimed that many practitioners may unnecessarily supplement P4 derivates due to significant individual variation in serum P4 concentrations [15] or because the diagnosis of true hypoluteoidism is difficult to establish. One aspect may also be that owners of pregnant bitches may be reluctant to regularly measure P4 concentrations until they decline, but rather are willing to supplement the bitches right away [17]. Despite the possibility of supplementing bitches affected by hypoluteoidism, more resorptions and generally smaller litter sizes in these cases have been described [20]. Further knowledge about the physiological range of P4 concentrations might be helpful in suspected cases of hypoluteoidism.

Recommendations for diagnostic approaches and treatment of suspected canine hypoluteoidism cases in practice vary in the scientific literature. According to some authors, blood samples should be drawn weekly, beginning five to seven days after last breeding [21]. Bitches should be supplemented if P4 decreases below 5.0 ng/mL before the last week of pregnancy [19,21]. Others initiated a supplementation when P4 concentrations below 10.0 ng/mL were measured at the 4th to 5th week of pregnancy and signs of impending abortion were observed [6]. A detailed guideline for P4 supplementation can be found in a publication by Becher et al. (2010) [15]. According to Becher and colleagues, P4 concentrations should be higher than 20.0 ng/mL between days 10 and 30, higher than 5.0 ng/mL between days 30 and 45 and higher than 1.5 ng/mL between days 45 and 58. Furthermore, the authors suggest that a sudden decline in P4 by 10 to 15 ng/mL between days 20 and 35 is indicative for hypoluteoidism and an indication for supplementation. The authors do not specify whether the days are counted from ovulation, LH surge or other. Other authors initiated supplementation of P4 if serum concentrations decreased by 20–30 ng/mL within 5 days [14].

The aim of this study was to assess the P4 concentrations in all stages of canine pregnancy using a large number of healthy dogs.

## 2. Materials and Methods

### 2.1. Animals

In total, 126 privately owned bitches of 65 different breeds were sampled in the course of this study. To be included, bitches had to be generally healthy within the past 6 months and receive no medication during that time. Previous gynecological illnesses were looked for but not present in any of the bitches. It was also expected of the bitches to be mated, with refusal resulting in exclusion from the study. Furthermore, cycle length and regularity, progress of previous pregnancies and litter sizes in those pregnancies, and possible hormone treatments were investigated. Informed written consent and approval from the owners was obtained (Animal testing approval number O 0095/18). Dogs were enrolled from March 2018 until December 2019.

### 2.2. Study Design

A first presentation of the bitches in the clinic took place to determine the day of ovulation. Provided they met the criteria to be included in the study, the initial examination before mating or insemination included an inspection of the vaginal cavity to assess the vaginal mucosa. During the inspection via speculum (Proctovision, Karl Storz, Tuttlingen, Germany) a vaginal smear was taken, stained and evaluated for the percentage distribution of vaginal epithelial cells and the potential occurrence of neutrophils. Furthermore, a general clinical assessment was performed, recording current body weight, rectal temperature, respiratory and heart rate. Finally, a blood sample for a comprehensive hematological evaluation and the determination of serum P4 concentration was obtained. Most bitches were repeatedly presented at the clinic until ovulation, which was defined as occurring when P4 reached concentrations between 4 and 8 ng/mL.

**Figure 1:**
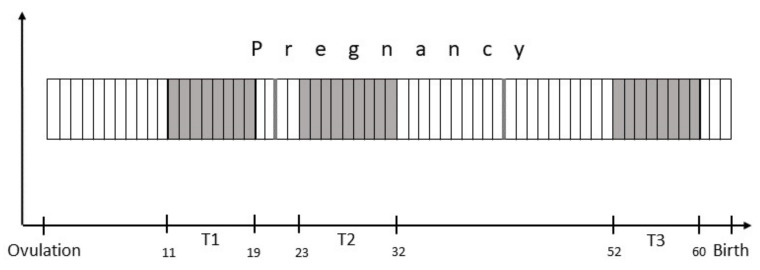
Scheduling of examinations T1 to T3 over the course of pregnancy.

For the purpose of this study, the three stages of canine pregnancy were defined as ranging from conception until day 21, from day 22 until day 42 and day 43 until parturition. As the protocol required one examination in each stage of pregnancy (see Figure 1), the bitches were next examined between day 11 and day 19 after ovulation (T1). This examination only included the general clinical assessment and the acquisition of a blood sample for serum P4 concentrations.

The next appointment took place between day 23 and day 32 (T2). The T2 examination included an ultrasonic examination to ascertain whether the bitch was pregnant. This examination was scheduled prior to the middle of the second third, to respect the owners’ wishes of having a pregnancy diagnosis sooner rather than later, while still completing the study examination within the same appointment. Again, the general clinical assessment was performed, and a blood sample was drawn. Bitches that had an inconclusive ultrasound result were reexamined a few days later. All bitches that had not become pregnant were assigned to the non-pregnant group (*n* = 25).

At the last examination between day 52 and 60 (T3), an ultrasound was also performed to check on the fetuses and the progress of the pregnancy, after the general clinical assessment and the extraction of blood.

The collection of all blood samples took place in the morning or early afternoon.

At parturition, the date of birth and thus the length of pregnancy from the time of ovulation and from first mating were recorded, as well as the type of delivery (natural or cesarean section). Furthermore, the number and weight of puppies (alive and stillborn) and survival after three weeks were documented, although the accuracy of this data is entirely dependent on the owner’s disclosure.

### 2.3. Blood Sampling and Sample Processing

Blood samples were collected by venipuncture from either the cephalic or the saphenous vein into plastic tubes (Sarstedt Tube 4.5 mL, Clotting Activator/Serum, 75 × 13 mm, Sarstedt AG & Co KG, Nümbrecht, Germany) and on the first examination also in an EDTA tube (EDTA KE/3ml, Sarstedt AG & Co KG, Nümbrecht, Germany). Samples were left at room temperature for 30 to 60 min for clotting and then centrifuged at 5000 rpm or 2884 g for 5 min (Hettich Centrifuge EBA 20, Hettich, Tuttlingen, Germany). Serum was transferred into a serum tube (Simport Cryovial sterile with lip seal design, external threads, 2 mL Tubes, Boloeil, QC, Canada) which was either placed in the refrigerator at 5.0 °C for a maximum of 4 h and then delivered to the laboratory nearby on the campus (LABOKLIN GmbH & Co. KG Steubenstr. 4, 97688 Bad Kissingen, Germany), or it was delivered to the laboratory at once.

The P4 concentrations were determined via chemiluminescence (Immulite 2000 XPi, Siemens Healthcare GmbH, Erlangen, Germany). Detection ranges were 0.20 ng/mL to 40.00 ng/mL. Concentrations larger than 40.00 ng/mL were set at 40.00 ng/mL and below 0.20 ng/mL at 0.20 ng/mL for statistical analyses. Validation for canine serum samples showed an intra-assay coefficient of variation (CV) of 3.72 to 4.77% and an inter-assay coefficient of variation (CV) of 2.72 to 3.32%.

### 2.4. Statistical Analysis

All data are expressed as the mean ± standard deviation or mean and range (min–max). For all statistical analyses, Microsoft Excel (Windows 10; Microsoft Corporation, Redmond, WA, USA) and SPSS (IBM SPSS Statistics 25; IBM Deutschland GmbH, Ehningen, Germany) were used. Differences in litter size and gestation length were compared between weight, age, parity and breed groups using a one-way ANOVA. Analyses of P4 concentrations were stratified by timepoint, to ensure that each bitch only occurred once in each analysis. P4 concentrations were compared between pregnancy status, weight and breed groups using a one-way ANOVA. A *p*-value ≤ 0.05 was considered significant.

## 3. Results

### 3.1. General Information

Of the 126 dogs enrolled, three bitches were excluded in the course of the study, as one bitch was ovariohysterectomized due to pyometra, one bitch was hypothyroid and had to be supplemented, and the owner of one bitch no longer wished to take part in the study. Hence, 123 bitches were eligible for further analysis, of which 25 did not become pregnant. Thus 20% of bitches did not become pregnant, although they were mated. In all bitches that did not become pregnant, ultrasound results were unremarkable, showing no signs of resorption. In addition, the bitches showed no further symptoms, such as abnormal vaginal discharge.

Inquiry concerning the reproductive history and previous hormonal medication in the bitches did not reveal any significant information in 117 of the bitches. Of the remaining six bitches, two were treated with aglepristone more than one year before enrollment because of undesired mating, two were treated with cabergoline, one for estrus induction more than a year previously (albeit this bitch came into estrus spontaneously this time) and one to treat symptoms of pseudopregnancy; one had developed a mild pyometra after the previous heat, which was treated successfully with antibiotics, and the 6^th^ was suspected to be suffering from mild subinvolution of placental sites after the previous birth, but recovered without medication. A total of seven bitches had previously undergone a cesarean section, with one elective and the remaining six due to dystocia.

The most common breeds in this study were French bulldogs (*n* = 8), miniature bull terriers (*n* = 7) and miniature smooth-haired dachshunds (*n* = 4), standard wire-haired dachshunds (*n* = 4), golden retrievers (*n* = 4) and English cocker spaniels (*n* = 4).

Body weights and age at the first examination ranged from 2.4 kg to 71.4 kg (mean 21.7 kg) and 15 months to 8 years (mean 4 years), respectively. All dogs were kept in their owners’ care during the study and were fed various commercial dog foods. Of the 123 bitches, 37 had had one previous litter, 22 had whelped two or more times, and 63 had had no previous litters. In one bitch, the parity was unknown. For detailed information, see Appendix A.

The average weight gain during pregnancy was 4.1 kg, with one bitch gaining as little as 600g and one bitch gaining 12.8 kg. The average relative weight increase was 21.6%, with one bitch gaining 3.6% and one bitch gaining 47.2% of their respective body weights.

Overall, 100 bitches were mated naturally. The remaining 23 bitches were artificially inseminated via a transcervical endoscopy using fresh semen (*n* = 16), frozen-thawed semen (*n* = 5) or chilled semen (*n* = 2). The reasons for the artificial inseminations were unwillingness of the bitch to be mated (*n* = 8), inexperienced males (*n* = 7), the use of semen from desirable males located too far away (*n* = 7) or concern of the owner regarding the males’ wellbeing (*n* = 1).

### 3.2. Delivery and Litter Size

In total, 83 bitches delivered naturally on days 57 to 66 after the first day of mating (mean 61) or on days 59 to 67 after ovulation (mean 63). Due to dystocia, 15 bitches delivered via C-section on days 58 to 64 after the first day of mating (mean 61) or days 61 to 68 after ovulation (mean 63). No elective C-sections were performed on any of the bitches in this study.

Gestation lasted 63 days after ovulation on average (59 to 68 days), with 23.4% of bitches whelping after exactly 63 days. Calculating the accuracy of prediction of the day of parturition using pre-breeding serum P4 concentrations yielded a 68.8% accuracy for day 63 ± 1, 92.2% for ±2, 94.8% for ±3 and 98.7% for ±4, while 100% accuracy was obtained for 63 ± 5 days.

The number of puppies born ranged from one puppy (*n* = 4) to 13 puppies (*n* = 5), with the mean number being six puppies. An overall number of 620 puppies were born, out of which 43 were stillborn and another 24 deceased in the first three weeks after birth.

The following parameters had an effect on litter size. Average litter size increased with the body weight of the bitch (see Table 2). The differences between the groups were significant (*p* ≤ 0.001). To be able to compare these results with those published in other articles, another evaluation was conducted using different weight categories: in bitches with a body weight of less than 10 kg (*n* = 24), gestation lasted 62.3 ± 1.2 days, in 10 to 25 kg bitches (*n* = 40) 63.1 ± 1.3 days, in 25 to 40 kg bitches (*n* = 26) 63.1 ± 1.6 days and in bitches heavier than 40 kg (*n* = 8) 63.0 ± 1.5 days.

Age had a significant effect on litter size (*p* = 0.03). With an average of eight puppies, the peak of litter size was reached by bitches of three to four years (see Table 2).

Bitches who had had no previous litters (*n* = 46) or one previous litter (*n* = 31) whelped seven puppies on average (one to 13 and three to 12, respectively). The average litter size in bitches who had had two (*n* = 14) previous litters was six (one to 12 puppies). Bitches with three or four previous litters (*n* = 6) whelped three puppies (two to eight).

To enhance comparability, the bitches were also sorted into breed groups according to the classification of the American Kennel Club (i.e., Herding, Hound, Non-Sporting, Sporting, Terrier, Toy, Working). Average litter size varied from three puppies in terrier breeds to eight puppies in sporting and working breeds.

Effects on gestation length were markedly different. No difference in the duration of gestation depending on body weight was evident, and neither age nor parity had a significant effect (see Table 2). Breed group did, however, influence gestation length. With 61.6 days, gestation length was shortest in toy breeds, and it was longest in terriers, with 63.7 days. Furthermore, litter size had a significant effect on gestation length (*p* = 0.03). With 62.4 days, it was shortest in large litters of nine to 13 pups, and significantly longer in litters of one to five pups, with 63.3 days.

Comparing the results of the T2 ultrasound to the actual litter size at birth showed that 72 of the 98 bitches were estimated correctly with a range of ± 1 puppy. More than half of these bitches (*n* = 36) were estimated with total accuracy.

### 3.3. Progesterone Analysis

A total of 366 samples were collected during pregnancy, as three non-pregnant bitches each missed one of the three appointments.

**Figure 2:**
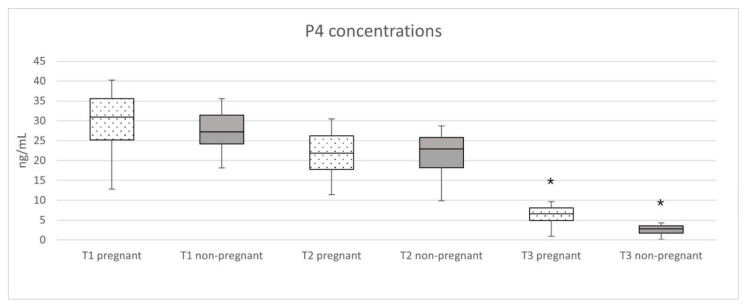
P4 concentrations of pregnant and non-pregnant bitches at the three examinations (T1 = d11–d19, T2 = d23–d32, T3 = d52–d60 post-ovulation) during pregnancy, with the asterisk (*) marking the examination with a significant difference among the groups. Each box displays the median P4 concentration as the thick line in the middle, with the upper and lower quartiles representing the limits of the box. The whiskers show the minimum and maximum values.

Mean serum P4 concentrations and distribution of concentrations are displayed in Figure 2. At the examinations during the first and second third, the mean serum P4 concentrations did not differ between the pregnant and the non-pregnant dogs. Only the last examination showed a significant (*p* ≤ 0.001) difference, with the pregnant bitches exhibiting higher P4 concentrations than the non-pregnant ones.

The P4 concentrations at T1 were 30.23 ± 6.65 ng/mL in the pregnant dogs and 28.45 ± 6.26 ng/mL in the non-pregnant ones. In the second third (T2) the concentrations were lower: 22.73 ± 6.27 ng/mL in the pregnant animals and 22.59 ± 5.77 ng/mL in the non-pregnant group. In the last third of pregnancy (T3) concentrations were 6.68 ± 2.18 ng/mL in the pregnant and 3.17 ± 2.26 ng/mL in the non-pregnant dogs. At the last examination, six non-pregnant bitches had P4 concentrations of <2 ng/mL. However, these bitches all had sufficiently high P4 concentrations at the previous two examinations, with only two bitches showing concentrations significantly below the mean for non-pregnant bitches. Those two bitches were one German shepherd dog and one Azawakh.

Taking a closer look at the P4 concentrations in relation to the sizes of the dogs, no differences were found between the subgroups at examinations T1, T2 and T3, respectively (see Table 3).

Grouping the bitches according to breed groups as described in 3.2. showed that terriers and non-sporting breeds in particular had a tendency for higher overall P4 concentrations during pregnancy or diestrus, while especially herding and hound breeds, but also sporting breeds, exhibited lower concentrations (see Table 4). This difference was significant at T2 and T3 (*p* = 0.002 and *p* ≤ 0.001).

**Figure 3:**
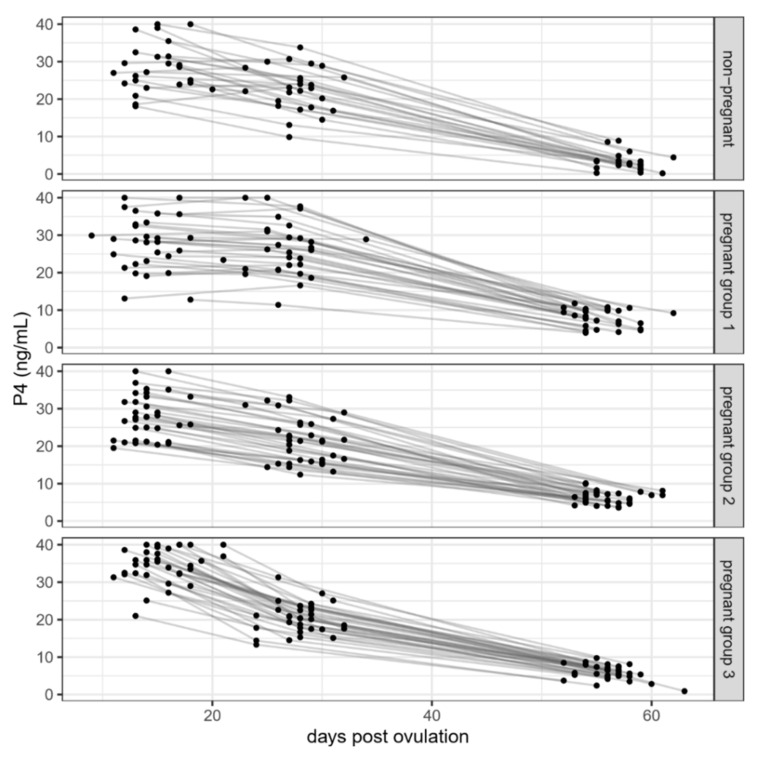
Course of the individual bitches’ P4 concentrations at the three examinations (T1 = d11–d19, T2 = d23–d32, T3 = d52–d60 post-ovulation). Animals were grouped according to pregnancy status. Pregnant animals were further grouped according to the extent of the decrease in P4 concentrations between timepoints T1 and T2 (group 1 decrease of <0.3 ng/mL per day, group 2 decrease between 0.3 and 0.7 ng/mL, group 3 decrease of >0.7 ng/mL).

Furthermore, the progression of P4 concentrations from the first to the second appointment was evaluated. Of the pregnant bitches, 28 showed a decline of more than 10 ng/mL from T1 to T2, with P4 concentrations decreasing by more than 15 ng/mL in 14 of the 28. All these bitches had a normal pregnancy and showed no signs of abortion or distress. For better visual separation, pregnant animals were further grouped according to the extent of the P4 decrease timepoints between T1 and T2. Individual developments of P4 concentrations can be examined in Figure 3.

Moreover, P4 concentrations before day 58, that is, at T3, were examined more closely. Of the 80 pregnant bitches that were examined before day 58 after ovulation, 18 had P4 concentrations lower than 5.00 ng/mL, but continued their pregnancy for four to 13 days before whelping.

## 4. Discussion

In this study, we assessed P4 concentrations during canine pregnancy using a large number of healthy dogs to supplement the existing knowledge. Several research groups have examined P4 concentrations in pregnant dogs, albeit with mostly small sample sizes and a limited number of breeds, so that the normal range of P4 concentrations still needs to be further assessed at different stages of the canine pregnancy.

A meta-analysis of 12 studies, published in 2012 [36], described data of a total number of 109 bitches, with beagles and Labrador retrievers accounting for roughly 80% of subjects, the remaining being German shepherd bitches (*n* = 11), cross-bred (*n* = 8) or unknown breeds (*n* = 4). As described in Section 1, a recent review of the existing literature by the authors of this study likewise revealed an overrepresentation of beagles and Labrador retrievers, and small total numbers of bitches studied. In that regard, it is warranted to conduct more studies with more dogs.

With the highest mean P4 concentrations of 29.87 ng/mL (30.23 ng/mL pregnant and 28.45 ng/mL non-pregnant) at T1 and lower mean P4 concentrations of 22.70 ng/mL (22.73 ng/mL pregnant and 22.59 ng/mL non-pregnant) at T2, the presented data are in accordance with some previously published results [32,33,37]. However, they contradict the findings of several other researchers, who stated that the highest overall P4 concentrations are reached between days 20 and 35 post ovulation [35,38,39]. Only 13 bitches (nine pregnant, four non-pregnant) reached the highest P4 concentrations at T2 with an increase of 0.70 to 4.50 ng/mL compared to T1, while the median decline in P4 in the other 110 bitches was 7.60 ng/mL (0.20 to 20.20 ng/mL). There was no noticeable similarity within this group of bitches when analyzing other factors assessed in this study. Considering the large individual variations of P4 concentrations demonstrated by many researchers [5,24,28], it is possible that the highest P4 concentrations would have been measured at a later date, if sampling had been performed daily or every few days.

The similarity of the pregnant and non-pregnant P4 concentrations at T1 and T2 agrees with most of the published literature, describing no relevant differences [4,13,32].

The significant difference in P4 concentrations between pregnant and non-pregnant bitches at T3 was a relevant finding. In six non-pregnant bitches, P4 concentrations had decreased below 2.00 ng/mL at T3 (days 52 to 60 after ovulation). Three bitches missed the third appointment. The other 16 non-pregnant bitches had a mean P4 concentration of 3.39 ± 2.03 ng/mL with individual concentrations as high as 8.60 ng/mL. Luz et al. [32] also measured lower mean P4 concentrations in non-pregnant compared to pregnant bitches in the last stage of pregnancy. Furthermore, they detected P4 concentrations below 1 ng/mL on average at day 66 in non-pregnant bitches, only three days later than in pregnant bitches. Concannon et al. [4] stated that a drop below 1 ng/mL in non-pregnant dogs occurred at days 51 to 82, which underlines the high variability of luteal regression. While new insights into the formation and function of canine corpora lutea have been studied, the mechanisms of luteal regression in the absence of pregnancy are still not fully understood [40].

The presented results show significant differences between breed groups in the second and last third of pregnancy. As highlighted in Section 3.3., the herding, hound and sporting breeds showed lower concentrations of P4 than the other breed groups throughout all appointments (see Table 4). Non-sporting and terrier breeds showed overall higher P4 concentrations than the other breed groups. As there is a paucity of breed-specific studies on P4 concentrations during pregnancy, no definite conclusions can be drawn on account of the presented results. Nonetheless, the knowledge of a tendency for lower P4 concentrations during pregnancy in certain breed groups may be helpful in cases of suspected hypoluteoidism, and should be investigated further.

Different proposals on how to manage suspected hypoluteoidism and when to start supplementing bitches have been published. Becher et al. (2010) [15] stated that P4 concentrations should be above 20 ng/mL between days 10 and 30 after ovulation. In this study, six pregnant bitches showed P4 concentrations lower than 20 ng/mL at T1. Of these bitches, five belonged to either the hound group or the sporting breed group. At T2 this number rose to 33 bitches, who all continued pregnancies without supplementation and whelped between one and thirteen puppies without an increased occurrence of complications. Of these bitches, 23 belonged to either the herding, hound or sporting breed group. Comparing the results of the T2 ultrasound to the litter size, only seven of the 33 bitches were estimated to give birth to more puppies.

The decline in P4 concentrations from the first to the second examination during pregnancy, which supposedly should not be larger than 10 to 15 ng/mL [15], was also evaluated. This suggestion is also not supported by the presented data. Of the pregnant bitches, 28 showed a decline of more than 10 ng/mL (average 15.15 ng/mL), continuing pregnancy without complications and overall whelping 163 puppies naturally, with 12 stillborn and nine puppy losses during the first three weeks after birth, and 27 puppies being born via cesarean section with five stillborn and one deceased. In this case, 18 of these bitches belonged to either the herding, hound or sporting group. Of the 28 bitches with a decline of more than 10 ng/mL, 14 showed a decline of more than 15 ng/mL. 12 of these bitches also had a normal pregnancy and whelped 83 puppies naturally with five stillborn and seven deceased. The remaining bitches had cesarean sections, with 14 healthy puppies and one stillborn. Of these bitches, nine belonged to either the herding, hound or sporting breed group. Again, comparing the T2 ultrasound results to the litter size at birth, only six of the 28 bitches were estimated to have a larger litter. The 70 bitches that showed a P4 decline of less than 10 ng/mL, or a rise in P4, whelped 430 puppies with 26 stillborn and 14 deceased. There were no clinically relevant differences in mean litter size or number of stillborn and deceased puppies between the bitches with “normal” P4 concentrations and those with concentrations lower than defined as normal. All non-pregnant bitches exhibited P4 concentrations sufficiently high to maintain a pregnancy at T1 and T2, and only six had P4 concentrations <2 ng/mL at T3. As the length of diestrus can vary in one bitch, depending on whether she is pregnant or not, it was assumed that these bitches were not suffering from hypoluteoidism but did not become pregnant for other reasons. The finding that 20% of bitches did not become pregnant is in accordance with average pregnancy rates after a combination of natural mating and insemination [41,42]. This proportion therefore did not raise suspicion that a high number of the included non-pregnant bitches reabsorbed due to pathological conditions. As no abnormal findings were observed in these bitches at the clinical and ultrasound examinations, and other parameters, such as the semen quality of the stud dogs, were widely unknown, no further diagnostic procedures were initiated in these cases.

Other authors [21] suggest that P4 concentrations should be higher than 5.00 ng/mL until day 58 of gestation. In this study, 18 of the 80 pregnant bitches examined before day 58 after ovulation had P4 concentrations lower than 5.00 ng/mL with an average of 4.19 ng/mL (2.39 to 4.95 ng/mL). Two bitches gave birth four days later (P4 concentrations 4.83 and 3.77 ng/mL respectively), the others were five to 13 days before parturition (mean eight days). Of these bitches, 14 belonged to either the herding, hound or sporting breed group, and only four had been estimated to give birth to more puppies.

Considering that supplementation of P4 can entail considerable risks for the bitch and the puppies, such as dystocia, pyometra and resultant septicemia, masculinization of female fetuses, fetal abnormalities, or fetal death [1,9], it is unsurprising that most authors caution against its use without a firm diagnosis. As the presented findings show that allegedly low P4 concentrations do not necessarily demonstrate an inability to maintain a pregnancy, it seems advisable to monitor the individual P4 progression more regularly and to consider possible breed-specific differences. Root-Kustritz (2001) [21] and Becher et al. (2010) [15] also propose a weekly blood sample, unless P4 concentrations drop below 10 ng/mL, at which point sampling should be performed two to four times a week.

Most studies where side effects have been observed after the administration of progestins were designed to examine long-time toxicity [43]. In their review from 2003, Romagnoli et al. [43] state that the occurrence of side effects is thought to be less likely in healthy adult bitches undergoing short-term treatment, but this conclusion is drawn mainly on empirical grounds. In three studies in which bitches expected to be suffering from luteal insufficiency were supplemented, an increased rate of c-sections and stillborn puppies were documented, although only one puppy showed congenital malformations [6,9,10]. Whether these findings were due to the supplementation remains open.

In a publication by Günzel-Apel at al. [13], the authors conclude that German shepherd dogs (*n* = 41) showed significantly lower P4 concentrations during diestrus and possibly during pregnancy than dogs of other breeds. This hypothesis can be supported by the data in this study, although we enrolled only three German shepherd bitches, out of which two became pregnant. At T1, the P4 concentrations were 20.40 ± 1.74 ng/mL for German shepherd bitches, and 29.60 ± 6.64 ng/mL for other bitches. At T2 and T3, similarly lower concentrations were detected with 14.40 ± 2.91 ng/mL vs. 22.20 ng/mL ± 6.17 ng/mL and 4.04 ± 2.42 ng/mL vs. 6.04 ng/mL ± 2.57 ng/mL. However, the number of German shepherds in the presented study was low and did not allow specific conclusions. Nevertheless, these data might be included in future reviews or meta-analyses.

The upper detection limit for measuring P4 concentrations was 40.0 ng/mL. Higher concentrations in 12 bitches (10 pregnant, two non-pregnant) at T1 and in two pregnant bitches at T2 could not be determined precisely. However, it is unlikely that a more accurate determination would have yielded biologically significant different findings.

In this study, the length of gestation ranged from 59 to 68 days (mean 63) and the accuracy of prediction of the day of parturition was 68.8% for 63 ± 1, 92.2% for ±2, 94.8% for ±3 and 98.7% for ±4 days, while 100% accuracy was reached for 63 ± 5 days. These results are comparable to other previously published findings [44,45].

Taking a closer look at the effect of litter size on gestation length, the interval from estimated ovulation to parturition averaged 63.3 ± 1.6 days for litters of one to five pups (*n* = 39), 62.7 ± 1.2 days for litters of six to eight pups (*n* = 40) and 62.4 ± 1.3 days for litters of nine to 13 pups (*n* = 19). The differences between the groups were significant (*p* = 0.03). These results are in accordance with those published by Tsutsui et al. (2006) [44]. Other authors chose to emphasize the difference between small and large litters by isolating singleton litters and litters of two puppies [46]. Applying this strategy to the presented results revealed more pronounced differences: for those litters with one or two pups (*n* = 6), the average length of gestation was 64.6 ± 1.4 days, while litters of three to nine pups (*n* = 62) and ten or more pups (*n* = 9) lasted 62.8 ± 1.3 and 62.4 ± 1.4 days, respectively. These findings are in accordance with those of Mir et al. (2011) [46]. Moreover, the authors state that bitches weighing less than 10 kg had a shorter mean gestation length than heavier bitches. This hypothesis was not confirmed by the results in this study: in bitches with a body weight of less than 10 kg (*n* = 24), gestation lasted 62.3 ± 1.2 days, while in bitches weighing 10 to 25 kg (*n* = 40), 25 to 40 kg (*n* = 26) or more than 40 kg (*n* = 8), gestation duration was 63.1 ± 1.3, 63.1 ± 1.6 and 63.0 ± 1.5 days, respectively. As in previously published work, age had no effect on the duration of gestation [47].

Additionally, as described in the study of Gavrilovic et al. (2008) [48], differences in litter size depending on the parity of the bitch were revealed. Bitches who had had no previous litters, one litter or two litters whelped six puppies on average, while bitches with three or four previous litters whelped three puppies. ANOVA showed a significant difference (*p* = 0.05) between bitches with less than or equal to two litters (*n* = 91) compared to bitches with three or four litters (*n* = 6). However, the large disparity between the two group sizes hardly allows for a conclusive inference. The age distribution showed a positive effect of advanced age on litter size until four years, then the average number of puppies declined. While bitches younger than two years whelped five puppies on average, and bitches of two to three years whelped six, bitches of three to four years had the largest mean litters with eight puppies. In older bitches, the number of puppies receded to a mean of six. The differences between the age groups were significant (*p* = 0.03). This finding coincides with previous publications [48,49]. An increase of average litter size with rising body weight in the bitch, as observed in this study (*p* ≤ 0.001), has also been described by other researchers [49].

The average relative weight gain of pregnant bitches of 22% was considerably lower than other published results [50]. This is probably due to the large number of different breeds studied and more variable litter sizes, although the breeds and number of animals or the litter sizes were not further specified in the cited publication.

Grouping the bitches by breed showed a statistically significant effect on litter size and gestation length. Notable is the fact that working breeds with the largest median litter size of eight had the second longest gestation length, with a mean of 63.4 days. Furthermore, the bitches of the toy breed group had the shortest mean gestation length of 61.6 days. These results are in contrast to those of Okkens et al. (2001) [51], where the smallest breed, West Highland white terriers, had the longest gestation duration of 62.8 days after mating. This difference may be due to the fact that there were only five pregnant bitches of toy breeds in this study, and they had comparably large litters of five puppies on average (three to six). As described in Section 3.2, bitches weighing less than or equal to 7.0 kg had the smallest mean litter size, but most of these bitches belonged to the hound breed group. Okkens et al. examined gestation length in different breeds in two studies [51,52] in 1993 and 2001. Terrier breeds (West Highland white terrier) had the longest gestation length of 62.8 days, followed by working breeds (Dobermann, boxer and Bernese mountain dog) with 61.8 days, herding breeds (German shepherd, old English sheepdog and Bouvier des Flandres) with 61.2 days and sporting breeds (Labrador retriever, golden retriever) with 60.9 days [51,52]. No hound, non-sporting or toy breeds were studied. However, Okkens et al. (1993 and 2001) calculated gestation length from the time of mating instead of from the estimated time of ovulation, so the data cannot be compared directly. As the time of mating was also based on the time of ovulation, which was determined after measuring P4 concentrations, two days can be added to the calculated gestation lengths to establish comparability. When employing this strategy, the previous data are in accordance with the results of this study, where terrier breeds gave birth after 63.7 days, working breeds after 63.4 days, herding breeds after 63.1 days and sporting breeds after 62.4 days. While the differences between the groups may not seem significant, further breed-specific knowledge will be useful when examining bitches close to parturition.

A limitation of the presented study was the use of privately owned bitches. Ideally, a comparative study would include strictly defined conditions of the study subjects with standardized surrounding factors, e.g., standardized feeding and housing, sampling on the same days of the cycle and standardized mating management. Our approach, nevertheless, enabled us to include a large number of bitches of various breeds, which better represent the dogs seen in everyday practice. The effect of feeding regimen on P4 has not yet been studied in the dog. Research on other species indicate potential effects, as high food intake induces higher P4 concentrations in pigs [53]. In ovariectomized rats, studies have shown that the simultaneous administration of estradiol and P4 resulted in decreased food intake [54,55]. The effect of food intake on P4 concentrations was not subject of this study, but reviewing the gathered data and available literature, it is indicated that feeding only has a minor effect. Since all samplings took place in the morning and early afternoon as indicated in Section 2.2, it is also unlikely that potential circadian secretion patterns, as have been described by some authors, have significantly influenced the presented results [5,56].

Furthermore, a study protocol with more frequent sampling could have yielded more specific results, but this would have significantly reduced the number of breeders willing to support the study, and thus reduced the power of the statistical analyses. Therefore, time frames during pregnancy were chosen that were feasible for the owners. The first examination (T1) was scheduled to assess P4 concentrations around the time of implantation, before the corpus luteum supposedly reaches its full potential [39]. The second examination (T2) enabled the evaluation of the progression of P4 concentrations during the prime of the corpus luteum, as many authors agree that the highest P4 concentrations are detected between days 20 and 35 post-ovulation [3,35]. Additionally, a drop in P4 concentrations due to luteal insufficiency may take place at days 25 to 35 of pregnancy in many cases of hypoluteoidism [17]. The third examination (T3) took place shortly before birth was expected, thus yielding P4 values during the late luteal phase [57].

## 5. Conclusions

Overall, the data presented in this study confirms the accepted progress of P4 concentrations during pregnancy. It is the first study on P4 concentrations during pregnancy with a large number of bitches and breeds studied. In the context of recent definitions of hypoluteoidism and interpretation of P4 concentrations at specific time points in the canine pregnancy, the gathered data do not support the previously published diagnosis and treatment recommendations. In total, 28 pregnant bitches showed a larger decline in P4 concentrations between T1 and T2, and 56 bitches showed P4 concentrations lower than the concentration recommended according to previous research findings. However, none of the animals in our study suffered from abortion or preterm delivery. Considering that supplementation of P4 can entail considerable risks for the bitch and the puppies, more research on P4 concentration patterns, diagnosis of hypoluteoidism and treatment indications and options is needed.

## Figures and Tables

**Table 1:** Literature review on previous publications about serum P4 concentrations in normal canine pregnancies. * German Shepherd, Rottweiler, Border Collie, Bernese Mountain Dog. *^1^ Golden Retriever, Irish Setter, Samoyed, English Bulldog, Hungarian Vizsla, Collie, Kerry Blue Terrier, Mastino. *^2^ Bernese Mountain Dog, Cavalier King Charles Spaniel.

Number (Breed)	Study Conclusion	Reference
6 (Beagle)	pregnant and non-pregnant P4 values were very similar	[22]
3 (Beagle)	P4 rose during estrus and remained increased for most of metestrus	[23]
9 (Beagle)	P4 peak at 20–25 days, then decreased until parturition	[24]
12 (Beagle)	P4 peak around day 25 after LH peak, declined from day 30 on	[4]
3 (Beagle, Crossbred)	P4 peak 18 days after mating, then declined	[25]
6 (German Shepherd, Greyhound)	no consistent P4 pattern in all bitches until 5th week, then gradually declining	[26]
3 (Labrador Retriever)	P4 peak within few days from estradiol peak, then decline (undetectable in one bitch 10 days before parturition)	[27]
8 (Beagle)	P4 peak day 14 after mating, decreased from day 35 on	[28]
7 (Labrador Retriever)	P4 peak at day 6, remaining elevated for 9 weeks, then declining	[29]
10 (Labrador Retriever, Beagle)	P4 remained high through 5–6 weeks, then declined	[30]
9 (Labrador Retriever)	highest P4 values from 2nd through 5th week, then declined	[31]
5 (Labrador Retriever)	diurnal differences from weeks 3 to 6; decline from week 3–4 on	[5]
8 (Crossbred)	P4 peak at days 18–20, declined from day 30 on	[32]
11 (German Shepherd)	P4 peak at days 5–15, then declined	[13]
5 (Beagle)	P4 peak at days 5–15, then declined	[33]
15 (breed not specified)	P4 peak in 4th week, then declined	[6]
12 (*)	P4 peak at days 12–18, then declined	[14]
14 (*^1^)	P4 peak in 2nd week, declined from 4th week on	[34]
12 (*^2^)	P4 19.2 ± 4.3/22.2 ± 3.9 ng/mL at days 23 to 29, 6.0 ± 1.3/8.7 ± 7.1 ng/mL at days 50 to 54, 4. ± 1.2/5.3 ± 2.8 ng/mL at days 55 to 59 in BMD and CKCS respectively	[35]

**Table 2:** Effects of different parameters (weight, age, parity, breed group) on litter size in dogs (expressed as median with range (min–max)) and gestation length (expressed as mean with range (min–max)).

Group	*n*	Litter Size	*p*-Value	Gestation Length (days)	*p*-Value
Weight			<0.001		0.13
≤7 kg	11	4 (2–6)		62.5 (60–64)	
7.1–14 kg	29	6 (1–10)		62.6 (59–65)	
14.1–32 kg	35	6 (1–10)		63.3 (60–68)	
>32 kg	23	9 (2–13)		62.8 (59–65)	
Age			0.03		0.22
<2 years	19	5 (1–12)		63.1 (61–67)	
2–3 years	20	6 (1–13)		62.7 (60–68)	
3–4 years	19	8 (3–13)		62.4 (59–65)	
>4 years	40	6 (1–13)		63.2 (59 -65)	
Parity			0.05		0.94
0–2 litters	91	6 (1–13)		62.9 (59–68)	
3–4 litters	6	3 (2–8)		62.9 (61–65)	
Breed group			0.01		0.02
Herding	14	5 (1–13)		63.1 (61–67)	
Hound	19	6 (3–13)		62.6 (59–65)	
Non-Sporting	15	7 (3–12)		62.7 (62–65)	
Sporting	15	8 (3–10)		62.4 (60–64)	
Terrier	17	3 (1–12)		63.7 (62–65)	
Toy	5	5 (3–6)		61.6 (60–63)	
Working	13	8 (2–13)		63.4 (61–68)	

**Table 3:** P4 concentrations at T1 = d 11–d19, T2 = d23-d32 and T3 = d52-d60 grouped by weight, expressed as mean ± standard deviation in ng/mL. Subjects for T1 and T2 included pregnant and non-pregnant bitches, while T3 only included pregnant bitches due to significant differences in P4 concentrations at T3 depicted in Figure 2.

Weight Group	T1	*n*	T2	*n*	T3	*n*
≤7 kg	30.67 ± 7.03	12	22.88 ± 4.82	12	6.12 ± 2.22	11
7.1–14 kg	31.82 ± 5.90	35	24.42 ± 7.04	35	6.85 ± 2.24	29
14.1–32 kg	28.57 ± 5.93	47	21.80 ± 5.50	47	6.95 ± 2.25	35
32.1–50 kg	29.81 ± 7.25	24	22.04 ± 7.07	24	6.24 ± 2.09	20
>50 kg	26.82 ± 11.82	5	21.80 ± 3.42	5	6.99 ± 2.22	3

**Table 4:** P4 concentrations at T1 = d 11–d19, T2 = d23-d32 and T3 = d52-d60, grouped by breed, expressed as mean ± standard deviation in ng/mL. Subjects for T1 and T2 included pregnant and non-pregnant bitches, while T3 only included pregnant bitches due to significant differences in P4 concentrations at T3 depicted in Figure 2.

Breed Groups	T1 (*p* = 0.10)	*n*	T2 (*p* = 0.002)	*n*	T3 (*p* ≤ 0.001)	*n*
Herding	27.27 ± 5.49	20	19.10 ± 4.37	20	5.26 ± 1.62	14
Hound	28.74 ± 7.46	23	20.72 ± 5.59	23	5.73 ± 2.01	19
Non-Sporting	32.85 ± 5.35	16	25.43 ± 6.01	16	7.98 ± 2.14	15
Sporting	28.52 ± 6.32	20	21.75 ± 5.82	20	6.60 ± 2.65	15
Terrier	31.83 ± 5.05	20	26.22 ± 5.73	20	7.71 ± 1.47	17
Toy	32.22 ± 4.36	6	23.77 ± 5.23	6	7.05 ± 1.60	5
Working	30.08 ± 8.02	18	23.59 ± 6.4	18	6.72 ± 2.25	13

## Data Availability

The data presented in this study are available in this paper and its Appendix A.

## Appendix A

**Table A1:** Supplementary information on the bitches enrolled in a study on progesterone concentrations in pregnancy or during diestrus.

Nr	Breed	Breed Group	Age (y)	Weight (kg)	Previous Litters	Previous Litter Sizes	Pregnant/Non- Pregnant	C- Section	Litter Size (incl. Stillborn)	♂	♀	Stillborn or	Deceased
001	Miniature Schnauzer	Terrier	2.5	6.3			NP						
002	Gordon Setter	Sporting	3.8	25.0			P		9	2	7		
003	Beagle	Hound	6.8	14.6	2	7/7	P		7	3	4		
004	English Cocker Spaniel	Sporting	5.9	13.2	4	8/5/2/5	P		3	3	0		
005	English Springer Spaniel	Sporting	2.9	20.4	1	6	NP						
006	Dogues des Bordeaux	Working	7.0	51.3	4	11/12/12/13	P		2	1	1		
007	Rottweiler	Working	3.1	43.6			P	CS	7	0	3	4	
008	Petit Brabançon	Toy	3.6	3.6	1	1	P		5	2	3		1
009	Standard Wire-Haired Dachshund	Hound	2.2	7.8			P		5	3	2		
010	Standard Wire-Haired Dachshund	Hound	5.0	5.6	2	5/5	P		4	2	2		
011	Dobermann	Working	4.2	38.8	1	3	NP						
012	Miniature Smooth Haired Dachshund	Hound	4.3	5.8	2	5/4	P		6	1	5		
013	Beagle	Hound	2.6	11.4			P		5	3	2		
014	Rhodesian Ridgeback	Hound	4.9	38.0	2	8/10	P		12	8	3	1	
015	Chihuahua	Toy	2.3	2.4	1	2	P		3	1	2		
016	Boxer	Working	2.9	28.8			P	CS	3	2	0	1	
017	Irish Terrier	Terrier	5.7	12.8	1	16	P		7	2	1	4	
018	Smooth Collie	Herding	1.3	19.6			P		5	3	2		1
019	Rhodesian Ridgeback	Hound	4.9	32.2			P		13	6	6	1	
020	Picardy Shepherd	Herding	2.2	23.5			NP						
021	Saarloos Wolfdog	Hound	6.2	28.8	2	5/6	P		5	2	3		
022	Standard Smooth Haired Dachshund	Hound	3.7	7.2	1	5	P		6	2	4		
023	Miniature Bull Terrier	Terrier	5.8	17.0	1	7	NP						
024	Miniature Australian Shepherd	Herding	3.3	12.2			NP						
025	Collie	Herding	6.3	24.0	2	7/1	P		5	2	3		
026	Cavalier King Charles Spaniel	Toy	3.7	7.0	1	3	P		4	2	2		
027	Dutch Sheepdog	Herding	5.0	14.4	1	7	P		6	4	2		
028	Borzoi	Hound	8.1	36.4			P	CS	8	4	4		
029	Miniature Wire-Haired Dachshund	Hound	1.6	4.2			P		4	3	1		
030	Miniature Poodle	Non-Sporting	1.9	7.6			P		4	2	2		
031	Hovawart	Working	3.4	35.8	1	10	P		12	4	8		
032	Miniature Wire-Haired Dachshund	Hound	6.9	5.5	4	6/6/6/6	P		6	5	1		
033	Jack Russell Terrier	Terrier	1.6	5.6			P		4	3	1		
034	Afghan Hound	Hound	4.1	24.4			NP						
035	Labrador Retriever	Sporting	2.6	22.4			P		9	7	2		
036	Vizsla	Sporting	3.6	24.0	1	9	NP						
037	Hovawart	Working	1.9	32.4			P		9	4	5		
038	German Shepherd	Herding	7.2	32.4	4	5/7/7/6	P		8	3	4	1	
039	Staffordshire Bull Terrier	Terrier	1.8	14.2			P		1			1	
040	French Bulldog	Non-Sporting	2.7	13.8			P		6	2	3	1	
041	Pyrenean Shepherd	Herding	5.5	13.0	2	5/1	P		1		1		
042	Standard Smooth Haired Dachshund	Hound	3.3	10.2	1	4	P		6	2	4		
043	French Bulldog	Non-Sporting	1.9	12.4	unclear		P		7	2	5		
044	Kromfohrlander	Non-Sporting	5.2	10.4	1	8	P		7	3	4		
045	French Bulldog	Non-Sporting	1.4	10.8			P		10	8	1	1	
046	French Bulldog	Non-Sporting	2.6	11.8	1	3	P		3	0	2	1	
047	Newfoundland Dog	Working	3.5	45.0			NP						
048	Standard Schnauzer	Working	7.8	18.2	1	5	P		6	3	3		
049	Miniature Wire-Haired Dachshund	Hound	6.1	5.5	3	5/5/6	P		3	2	1		
050	Old English Bulldog	Non-Sporting	3.0	28.4	1	11	P		9	5	3	1	
051	Parson Russell Terrier	Terrier	7.2	7.0	3	4/3/3	P		2	1	1		
052	German Shepherd	Herding	7.9	33.6	4	6/9/8/6	NP						
053	Old English Bulldog	Non-Sporting	2.0	32.2			P		12	6	5	1	
054	Miniature Bull Terrier	Terrier	4.7	19.0	1	8	P	CS	7	6	1		
055	Collie	Herding	6.1	29.6	1	8	NP						
056	Giant Schnauzer	Working	2.4	37.6			P		6	4	1	1	1
057	Tervuren	Herding	5.8	19.6	1	5	P		6	4	2		
058	Miniature Bull Terrier	Terrier	4.8	16.4	1	7	P		6	1	3	2	
059	Scottish Terrier	Terrier	5.5	8.8	1	1	P	CS	3	0	3		
060	Weimaraner	Sporting	3.4	30.8			P		8	3	5		
061	Cairn Terrier	Terrier	1.9	8.0			P		2	1	1		
062	Australian Shepherd	Herding	6.4	26.8			NP						
063	Affenpinscher	Toy	6.1	7.8	2	2/7	P	CS	6	1	4	1	
064	Standard Wire-Haired Dachshund	Hound	4.9	9.0	2	7/6	P		7	3	4		
065	Collie	Herding	1.6	22.0			P		2	1	1		
066	Vizsla	Sporting	4.0	26.4	1	8	P		10	5	5		1
067	Boxer	Working	6.8	27.8	1	6	P		4	1	3		2
068	Pug	Toy	2.9	7.6			NP						
069	Dobermann	Working	4.9	38.6	1	3	P		9	4	2	3	3
070	Jack Russell Terrier	Terrier	4.1	5.5	1	4	P		3	2	1		1
071	Kromfohrlander	Non-Sporting	2.3	9.2			P		9	7	2		
072	Hovawart	Working	6.7	41.6	2	10/12	P		8	3	4	1	
073	Malinois	Herding	4.1	21.6			P		4	0	4		
074	Nova Scotia Duck Tolling Retriever	Sporting	3.8	21.0	1	9	P	CS	8	4	3	1	
075	Old German Shepherd Dog	Herding	1.8	33.2			P		10	5	5		1
076	Irish Wolfhound	Hound	2.9	56.4			P		13	8	4	1	
077	Black Russian Terrier	Terrier	3.4	46.4	1	13	P		12	9	2	1	
078	Newfoundland Dog	Working	4.0	46.0			NP						
079	Golden Retriever	Sporting	4.4	22.8			P		5	2	3		
080	Golden Retriever	Sporting	3.8	33.6	1	13	P		8	5	3		
081	Entlebucher Mountain Dog	Herding	2.6	23.6			P		7	3	4		
082	Cairn Terrier	Terrier	1.9	8.2			P		7	3	4		
083	Boerboel	Working	4.0	71.4			NP						
084	Pug	Toy	2.1	8.8			P		5	2	3		
085	Cairn Terrier	Terrier	2.7	8.0			P		5	1	4		
086	Beagle	Hound	3.5	11.0	1	5	NP						
087	Miniature Bull Terrier	Terrier	1.8	14.6			P		3	2	1		
088	French Bulldog	Non-Sporting	3.1	13.6	1	8	P	CS	7	3	4		
089	Greater Swiss Mountain Dog	Working	2.0	48.0			P	CS	13	3	7	3	4
090	English Setter	Sporting	2.4	25.0			P		7	4	3		1
091	Polish Lowland Sheepdog	Herding	3.8	15.0	2	3/2	P		3	0	3		
092	Miniature Poodle	Non-Sporting	1.8	7.8			P		5	3	2		
093	German Shepherd	Herding	6.9	27.0			P	CS	1			1	
094	Dogues des Bordeaux	Working	3.3	49.0			P		13	7	6		
095	Golden Retriever	Sporting	4.6	33.0			P		6	2	4		
096	Miniature Bull Terrier	Terrier	5.4	16.0	2	6/5	P	CS	6	2	3	1	1
097	Eurasian	Non-Sporting	3.8	20.8	1	5	P		5	3	2		
098	Azawakh	Hound	7.2	19.0	1	8	NP						
099	Standard Wire-Haired Dachshund	Hound	3.4	9.2			P		6	2	4		
100	Nova Scotia Duck Tolling Retriever	Sporting	5.8	15.8	1	4	P		5	3	2		
101	Kromfohrlander	Non-Sporting	2.9	10.4			P		7	3	3	1	
102	English Cocker Spaniel	Sporting	2.9	11.2	1	8	P		8	6	2		
103	Golden Retriever	Sporting	2.6	32.8			P		8	4	4		
104	Labrador Retriever	Sporting	5.3	28.2	1	7	P		6	2	4		
105	English Cocker Spaniel	Sporting	2.9	11.2	1	8	P	CS	6	4	2		
106	Dutch Shepherd	Herding	3.4	38.8			P		13	5	8		
107	Spinone Italiano	Sporting	6.3	32.0			NP						
108	English Cocker Spaniel	Sporting	8.2	12.6	2	4/3	NP						
109	Smooth Collie	Herding	2.2	21.6			NP						
110	Black Russian Terrier	Terrier	7.1	54.0	2	5/5	P	CS	3	1	1	1	1
111	Entlebucher Mountain Dog	Herding	5.0	23.2	2	2/3	P		4	2	2		
112	Dogues des Bordeaux	Working	8.0	53.2			NP						
113	Azawakh	Hound	7.2	19.4			NP						
114	Miniature Bull Terrier	Terrier	2.3	12.0			NP						
115	English Setter	Sporting	3.0	26.4			NP						
116	Miniature Bull Terrier	Terrier	1.3	14.6			P		3	1	2		1
117	Azawakh	Hound	7.9	18.8	1	8	P		7	2	5		
118	Saarloos Wolfdog	Hound	4.5	30.4			P		6	2	4		3
119	French Bulldog	Non-Sporting	1.4	12.6			P		8	3	5		
120	French Bulldog	Non-Sporting	2.1	12.2			P		6	3	2	1	2
121	Greater Swiss Mountain Dog	Working	3.7	39.0	1	8	P	CS	10	2	5	3	
122	French Bulldog	Non-Sporting	1.8	10.4			NP						
123	Miniature Bull Terrier	Terrier	2.9	16.8			P	CS	6	1	2	3	

**Table A2:** P4 concentrations in ng/mL of all bitches at the three examinations (T1 = d11–d19, T2 = d23–d32; T3 = d52–d60).

Number	Pregnant/ Non-Pregnant	T1 P4	T2 P4	T3 P4
001	NP	23.00	24.00	4.44
002	P	35.30	30.90	9.99
003	P	35.50	23.60	4.95
004	P	28.2	20.4	4.17
005	NP	25.00	17.20	3.37
006	P	13.10	16.6	4.49
007	P	29.00	26.10	8.40
008	P	40.00	32.20	6.58
009	P	36.90	21.40	7.30
010	P	38.6	23.70	7.35
011	NP	25.1	19.50	3.40
012	P	19.90	21.00	4.15
013	P	24.80	16.40	3.58
014	P	12.8	11.40	3.93
015	P	32.50	28.20	9.64
016	P	21.10	13.2	4.78
017	P	36.10	22.50	6.48
018	P	31.90	21.10	4.15
019	P	32.10	17.80	5.78
020	NP	26.20	23.80	1.61
021	P	21.00	13.30	3.72
022	P	31.3	16.7	6.66
023	NP	31.40	28.90	6.02
024	NP	22.60	22.1	/
025	P	25.10	15.30	4.50
026	P	33.5	17.6	6.72
027	P	21.20	12.40	5.32
028	P	22.3	18.60	4.62
029	P	20.70	15.70	5.61
030	P	27.20	17.50	4.89
031	P	34.20	26.30	5.69
032	P	27.80	21.50	6.06
033	P	31.80	25.90	7.22
034	NP	35.50	30.00	4.87
035	P	19.50	14.40	6.43
036	NP	29.60	30.70	2.49
037	P	35.10	31.00	4.91
038	P	20.40	15.30	4.83
039	P	29.30	28.90	10.6
040	P	32.90	29.40	10.8
041	P	28.20	26.00	9.2
042	P	32.50	22.60	6.6
043	P	29.60	26.70	10.60
044	P	34.70	14.50	5.24
045	P	35.6	37.10	6.51
046	P	26.70	21.50	9.94
047	NP	27.00	28.40	3.56
048	P	40.00	20.4	8.48
049	P	40.00	25.10	0.90
050	P	39.0	24.20	9.75
051	P	27.8	16.60	5.58
052	NP	18.1	9.88	0.30
053	P	33.20	27.30	6.92
054	P	36.90	32.20	7.81
055	NP	29.10	21.80	3.54
056	P	40.00	27.00	8.10
057	P	29.00	22.80	5.14
058	P	27.70	21.40	7.49
059	P	33.20	29.00	6.91
060	P	19.10	20.70	4.15
061	P	30.60	21.70	8.11
062	NP	39.00	22.20	2.67
063	P	25.80	21.20	7.02
064	P	40.00	37.70	6.30
065	P	31.80	22.90	8.22
066	P	40.00	31.30	7.21
067	P	27.10	22.50	7.37
068	NP	32.50	24.90	/
069	P	35.70	20.30	5.62
070	P	32.40	23.10	7.51
071	P	40.00	33.10	7.16
072	P	40.00	40.00	11.80
073	P	25.90	23.80	4.93
074	P	35.90	19.30	5.36
075	P	34.40	17.40	3.49
076	P	24.40	24.10	7.77
077	P	38.00	20.90	5.06
078	NP	24.20	18.20	2.93
079	P	21.00	15.30	7.57
080	P	32.20	22.60	8.10
081	P	25.00	17.50	4.05
082	P	35.80	34.90	7.00
083	NP	38.60	20.20	0.2
084	P	29.00	18.50	5.27
085	P	37.50	40.00	9.85
086	NP	31.30	25.60	1.05
087	P	28.10	29.2	10.10
088	P	34.30	25.70	7.11
089	P	21.50	15.90	3.77
090	P	21.30	22.00	10.30
091	P	37.6	20.10	6.33
092	P	20.80	16.30	5.91
093	P	21.50	14.40	4.04
094	P	36.50	32.60	8.33
095	P	33.90	17.80	6.47
096	P	28.60	25.40	7.76
097	P	29.20	27.40	9.89
098	NP	24.40	14.50	0.40
099	P	19.80	19.60	5.81
100	P	29.90	31.00	10.70
101	P	40.00	20.80	5.52
102	P	40.00	24.20	2.83
103	P	25.40	26.20	8.56
104	P	24.90	20.80	4.74
105	P	32.4	14.40	2.39
106	P	29.60	15.1	4.87
107	NP	28.60	16.90	1.42
108	NP	20.90	13.10	2.45
109	NP	23.90	22.90	2.35
110	P	39.40	25.00	8.72
111	P	24.90	15.20	4.59
112	NP	18.60	23.10	/
113	NP	29.50	17.80	3.02
114	NP	40.00	33.80	8.57
115	NP	27.20	25.80	2.11
116	P	23.40	22.20	7.23
117	P	23.10	19.70	9.85
118	P	36.9	18.70	7.96
119	P	29.00	24.30	10.10
120	P	33.40	31.50	9.41
121	P	34.70	23.40	5.60
122	NP	40.00	29.50	8.90
123	P	25.60	18.80	7.63

## References

[1] Tsutsui T. (1983). Effects of ovariectomy and progesterone treatment on the maintenance of pregnancy in bitches. Jpn. J. Vet. Sci..

[2] Okkens A.C., Dieleman S.J., Bevers M.M., Willemse A.H. (1985). Evidence for the non-involvement of the uterus in the lifespan of the corpus luteum in the cyclic dog. Vet. Q..

[3] Verstegen-Onclin K., Verstegen J. (2008). Endocrinology of pregnancy in the dog: A review. Theriogenology.

[4] Concannon P.W., Hansel W., Visek W.J. (1975). The ovarian cycle of the bitch: Plasma estrogen, LH and progesterone. Biol. Reprod..

[5] Steinetz B.G., Goldsmith L.T., Hasan S.H., Lust G. (1990). Diurnal variation of serum progesterone, but not relaxin, prolactin, or estradiol-17β in the pregnant bitch. Endocrinology.

[6] Tibold A., Thuróczy J. (2009). Progesterone, oestradiol, FSH and LH concentrations in serum of progesterone-treated pregnant bitches with suspected luteal insufficiency. Reprod. Domest. Anim..

[7] Concannon P.W., Hansel W. (1977). Prostaglandin F2α induced luteolysis, hypothermia, and abortions in beagle bitches. Prostaglandins.

[8] Kowalewski M.P. (2012). Endocrine and molecular control of luteal and placental function in dogs: A review. Reprod. Domest. Anim..

[9] Görlinger S., Galac S., Kooistra H.S., Okkens A.C. (2005). Hypoluteoidism in a bitch. Theriogenology.

[10] Günzel-Apel A.R., Urhausen C., Wolf K., Einspanier A., Oei C., Piechotta M. (2012). Serum progesterone in pregnant bitches supplemented with progestin because of expected or suspected luteal insufficiency. Reprod. Domest. Anim..

[11] Johnson C.A. (2008). High-risk pregnancy and hypoluteoidism in the bitch. Theriogenology.

[12] Johnson C.A. (2008). Pregnancy management in the bitch. Theriogenology.

[13] Günzel-Apel A.R., Zabel S., Bunck C.F., Dieleman S.J., Einspanier A., Hoppen H.-O. (2006). Concentrations of progesterone, prolactin and relaxin in the luteal phase and pregnancy in normal and short-cycling German Shepherd dogs. Theriogenology.

[14] Krachudel J., Bondzio A., Einspanier R., Einspanier A., Gottschalk J., Kuechenmeister U., Muennich A. (2013). Luteal insufficiency in bitches as a consequence of an autoimmune response against progesterone?. Theriogenology.

[15] Becher A., Wehrend A., Goericke-Pesch S. (2010). Luteal insufficiency in the bitch—Symptoms, diagnosis, consequences and therapy. A review of the literature. Tierarztl. Praxis Ausg. K Kleintiere Heimtiere.

[16] Rosset E., Mazereaux C., Buff S. Retrospective study of hypoluteoidism cases in the bitch. Proceedings of the 2010 EVSSAR Congress, Luvain La Neuve.

[17] Verstegen J., Dhaliwal G., Verstegen-Onclin K. (2008). Canine and feline pregnancy loss due to viral and non-infectious causes: A review. Theriogenology.

[18] Graham E.M., Taylor D.J. (2012). Bacterial reproductive pathogens of cats and dogs. Vet. Clin. N. Am. Small Anim. Pract..

[19] Root Kustritz M.V. (2005). Pregnancy diagnosis and abnormalities of pregnancy in the dog. Theriogenology.

[20] Günzel-Apel A.R., Zabel S., Einspanier A., Hoppen H.O. „Significance, diagnostics and handling of luteal insufficiency in the dog: Breeders‘ wishes and the veterinarians‘ responsibility. ” In Proceedings of the 49th yearly conference of the section small animal diseases of the German Veterinary Society DVG.

[21] Root Kustritz M.V., Concannon P.W., England G., Verstegen J., Linde Forsberg C. (2001). Use of supplemental progesterone in management of canine pregnancy. Recent Advances in Small Animal Reproduction.

[22] Parkes M.F., Bell E.T., Christie D.W. (1972). Plasma progesterone levels during pregnancy in the beagle bitch. Br. Vet. J..

[23] Jones G.E., Boyns A.R., Cameron E.H.D., Bell E.T., Christie D.W., Parkes M.F. (1973). Plasma oestradiol, luteinizing hormone and progesterone during pregnancy in the Beagle bitch. Reproduction.

[24] Smith M.S., McDonald L.E. (1974). Serum levels of luteinizing hormone and progesterone during the estrous cycle, pseudopregnancy and pregnancy in the dog. Endocrinology.

[25] Hadley J.C. (1975). Total unconjugated oestrogen and progesterone concentrations in peripheral blood during pregnancy in the dog. Reproduction.

[26] Edqvist L.-E., Johansson E.D.B., Kasström H., Olsson S.-E., Richkind M. (1975). Blood plasma levels of progesterone and oestradiol in the dog during the oestrous cycle and pregnancy. J. Endocrinol..

[27] Austad R., Lunde A., Sjaastad O.V. (1976). Peripheral plasma levels of oestradiol-17 β and progesterone in the bitch during the oestrous cycle, in normal pregnancy and after dexamethasone treatment. J. Reprod. Fertil..

[28] Gräf K.-J. (1978). Serum oestrogen, progesterone and prolactin concentrations in cyclic, pregnant and lactating beagle dogs. Reproduction.

[29] Chakraborty P.K. (1987). Reproductive hormone concentrations during estrus, pregnancy and pseudopregnancy in the Labrador bitch. Theriogenology.

[30] Steinetz B.G., Goldsmith L.T., Lust G. (1987). Plasma relaxin levels in pregnant and lactating dogs. Biol. Reprod..

[31] Steinetz B.G., Goldsmith L.T., Harvey H.J., Lust G. (1989). Serum relaxin and progesterone concentrations in pregnant, pseudopregnant, and ovariectomized, progestin-treated pregnant bitches: Detection of relaxin as a marker of pregnancy. Am. J. Vet. Res..

[32] Luz M.R., Bertan C.M., Binelli M., Lopes M.D. (2006). Plasma concentrations of 13,14-dihydro-15-keto prostaglandin F2-alpha (PGFM), progesterone and estradiol in pregnant and nonpregnant diestrus cross-bred bitches. Theriogenology.

[33] Günzel-Apel A.R., Beste N., Nottorf S., Eschricht F., Hoppen H.O., Dieleman S., Einspanier A. (2009). Comparison of selected endocrine parameters during luteal phase and pregnancy in German Shepherd dogs and Beagles. Reprod. Domest. Anim..

[34] Thuróczy J., Müller L., Kollár E., Balogh L. (2016). Thyroxin and progesterone concentrations in pregnant, nonpregnant bitches, and bitches during abortion. Theriogenology.

[35] Thejll Kirchhoff K., Goericke-Pesch S. (2016). Changes in serum progesterone concentrations in Bernese mountain dogs and Cavalier King Charles Spaniels during pregnancy. Theriogenology.

[36] Sontas B.H., Speroni M., Stelletta C., Günzel-Apel A.R., Romagnoli S. Serum progesterone patterns in the pregnant bitch: A meta-analysis. Proceedings of the 7th International Symposium on Canine and Feline Reproduction.

[37] Hadley J.C. (1975). Total unconjugated oestrogen and progesterone concentrations in peripheral blood during the oestrous cycle of the dog. Reproduction.

[38] Concannon P.W. (2011). Reproductive cycles of the domestic bitch. Anim. Reprod. Sci..

[39] Papa P.C., Hoffmann B. (2011). The corpus luteum of the dog: Source and target of steroid hormones?. Reprod. Domest. Anim..

[40] Papa P.C., Kowalewski M.P. (2020). Factors affecting the fate of the canine corpus luteum: Potentital contributors to pregnancy and non-pregnancy. Theriogenology.

[41] Farstad W. (1984). Bitch fertility after natural mating and after artifical insemination with fresh or frozen semen. J. Small Anim. Pract..

[42] Baalbergen T. (2021). Ovulation Timing in the Bitch: Conception Rate and Influencing Factors in 1401 Estrus Cycles. Master’s Thesis.

[43] Romagnoli S., Concannon P.W., Concannon P.W., England G., Verstegen J., Linde Forsberg C. (2003). Clinical use of progestins in bitches and queens: A review. Recent Advances in Small Animal Reproduction.

[44] Tsutsui T., Hori T., Kirihara N., Kawakami E., Concannon P.W. (2006). Relation between mating or ovulation and the duration of gestation in dogs. Theriogenology.

[45] Kutzler M.A., Mohammed H.O., Lamb S.V., Meyers-Wallen V.N. (2003). Accuracy of canine parturition date prediciton from the initital rise in preovulatory progesterone concentration. Theriogenology.

[46] Mir F., Billault C., Fontaine E., Sendra J., Fontbonne A. (2011). Estimated pregnancy length from ovulation to parturition in the bitch and its influencing factors: A retrospective study in 162 pregnancies. Reprod. Domest. Anim..

[47] Eilts B.E., Davidson A.P., Hosgood G., Paccamonti D.L., Baker D.G. (2005). Factors affecting gestation duration in the bitch. Theriogenology.

[48] Gavrilovic B.B., Andersson K., Linde Forsberg C. (2008). Reproductive patterns in the domestic dog—A retrospective study of the Drever breed. Theriogenology.

[49] Borge K.S., Tønnessen R., Nødtvedt A., Indrebø A. (2011). Litter size at birth in purebred dogs—A retrospective study of 224 breeds. Theriogenology.

[50] Concannon P.W., McCann J.P., Temple M. (1989). Biology and endocrinology of ovulation, pregnancy and parturition in the dog. J. Reprod. Fertil. Suppl..

[51] Okkens A.C., Teunissen J.M., Van Osch W., Van Den Brom W.E., Dieleman S.J., Kooistra H.S. (2001). Influence of litter size and breed on the duration of gestation in dogs. J. Reprod. Fertil. Suppl..

[52] Okkens A.C., Hekerman T.W.M., De Vogel J.W.A., Van Haaften B. (1993). Influence of litter size and breed on variation in length of gestation in the dog. Vet. Q..

[53] Athorn R.Z., Stott P., Bouwman E.G., Chen T.Y., Kennaway D.J., Langendijk P. (2013). Effect of feeding level on luteal function and progesterone concentration in the vena cava during early pregnancy in gilts. Reprod. Fertil. Dev..

[54] Varma M., Chai J.-K., Meguid M.M., Laviano A., Gleason J.R., Yang Z.-J., Blaha V. (1999). Effect of estradiol and progesterone on daily rhythm in food intake and feeding patterns in Fischer rats. Physiol. Behav..

[55] Asarian L., Geary N. (2006). Modulation of appetite by gonadal steroid hormones. Philos. Trans. R. Soc. B Biol. Sci..

[56] Thuróczy J., Wölfling A., Tibold A., Balogh L., Jánoki G. (2003). A; Solti, L Effect of anticoagulants and sampling time on results of progesterone determination in canine blood samples. Reprod. Domest. Anim..

[57] Marinelli L., Rota A., Carnier P., Da Dalt L., Gabai G. (2009). Factors affecting progesterone production in corpora lutea from pregnant and diestrous bitches. Anim. Reprod. Sci..

